# Transcript profile of *CLSTN3B* gene in human white adipose tissue is associated with obesity and mitochondrial gene program

**DOI:** 10.1093/lifemeta/load037

**Published:** 2023-09-14

**Authors:** Ningning Bai, Xuhong Lu, Yansu Wang, Xiaoya Li, Rong Zhang, Haoyong Yu, Cheng Hu, Xiaojing Ma, Yuqian Bao, Ying Yang

**Affiliations:** Department of Endocrinology and Metabolism, Shanghai Diabetes Institute, Shanghai Clinical Center for Diabetes, Shanghai Key Laboratory of Diabetes Mellitus, Shanghai Key Clinical Center for Metabolic Disease, Shanghai Jiao Tong University School of Medicine Affiliated Sixth People’s Hospital, Shanghai 200233, China; Department of Endocrinology, Shenzhen Second People’s Hospital, the First Affiliated Hospital of Shenzhen University, Health Science Center of Shenzhen University, Shenzhen Clinical Research Center for Metabolic Diseases, Shenzhen Center for Diabetes Control and Prevention, Shenzhen, Guangdong 518000, China; Department of Endocrinology and Metabolism, Shanghai Diabetes Institute, Shanghai Clinical Center for Diabetes, Shanghai Key Laboratory of Diabetes Mellitus, Shanghai Key Clinical Center for Metabolic Disease, Shanghai Jiao Tong University School of Medicine Affiliated Sixth People’s Hospital, Shanghai 200233, China; Department of Endocrinology and Metabolism, Shanghai Diabetes Institute, Shanghai Clinical Center for Diabetes, Shanghai Key Laboratory of Diabetes Mellitus, Shanghai Key Clinical Center for Metabolic Disease, Shanghai Jiao Tong University School of Medicine Affiliated Sixth People’s Hospital, Shanghai 200233, China; Department of Endocrinology and Metabolism, Shanghai Diabetes Institute, Shanghai Clinical Center for Diabetes, Shanghai Key Laboratory of Diabetes Mellitus, Shanghai Key Clinical Center for Metabolic Disease, Shanghai Jiao Tong University School of Medicine Affiliated Sixth People’s Hospital, Shanghai 200233, China; Department of Endocrinology and Metabolism, Shanghai Diabetes Institute, Shanghai Clinical Center for Diabetes, Shanghai Key Laboratory of Diabetes Mellitus, Shanghai Key Clinical Center for Metabolic Disease, Shanghai Jiao Tong University School of Medicine Affiliated Sixth People’s Hospital, Shanghai 200233, China; Department of Endocrinology and Metabolism, Shanghai Diabetes Institute, Shanghai Clinical Center for Diabetes, Shanghai Key Laboratory of Diabetes Mellitus, Shanghai Key Clinical Center for Metabolic Disease, Shanghai Jiao Tong University School of Medicine Affiliated Sixth People’s Hospital, Shanghai 200233, China; Department of Endocrinology and Metabolism, Shanghai Diabetes Institute, Shanghai Clinical Center for Diabetes, Shanghai Key Laboratory of Diabetes Mellitus, Shanghai Key Clinical Center for Metabolic Disease, Shanghai Jiao Tong University School of Medicine Affiliated Sixth People’s Hospital, Shanghai 200233, China; Department of Endocrinology and Metabolism, Shanghai Diabetes Institute, Shanghai Clinical Center for Diabetes, Shanghai Key Laboratory of Diabetes Mellitus, Shanghai Key Clinical Center for Metabolic Disease, Shanghai Jiao Tong University School of Medicine Affiliated Sixth People’s Hospital, Shanghai 200233, China; Department of Endocrinology and Metabolism, Shanghai Diabetes Institute, Shanghai Clinical Center for Diabetes, Shanghai Key Laboratory of Diabetes Mellitus, Shanghai Key Clinical Center for Metabolic Disease, Shanghai Jiao Tong University School of Medicine Affiliated Sixth People’s Hospital, Shanghai 200233, China; Department of Endocrinology and Metabolism, Shanghai Diabetes Institute, Shanghai Clinical Center for Diabetes, Shanghai Key Laboratory of Diabetes Mellitus, Shanghai Key Clinical Center for Metabolic Disease, Shanghai Jiao Tong University School of Medicine Affiliated Sixth People’s Hospital, Shanghai 200233, China

## Dear Editor,

Obesity is a major health issue with global prevalence and increases the risk of many metabolic diseases. Of particular concern is the increasing incidence of type 2 diabetes, the primary causes of which are obesity-driven insulin resistance in white adipose tissue (WAT), skeletal muscle, and liver, and decreased insulin secretion by pancreatic β-cells [1]. Abnormal adipose expansion is accompanied by adverse consequences such as chronic inflammation and fibrosis, dysregulated adipokine secretion, and mitochondrial dysfunction [2]. Although many mole­cules are known to regulate adipose function, factors that are highly specific for WAT function and metabolic status remain underexplored.

Calsyntenin 3b (*Clstn3b*) is a novel gene that promotes brown fat thermogenesis in mice [3]. It is an abundant adipocyte-selective product of the mouse *Clstn3* locus, and has three exons: the first exon is large and unique and the last two are shared with the known *Clstn3* gene. We previously reported that the transcript form of *Clstn3*/*CLSTN3* locus in WAT is discordant between mice and humans. *CLSTN3* is routinely expressed in human white fat, while the form of *CLSTN3B* appears to be rare [4]. A recent study identified that *Clstn3b* enforces adipocyte multilocularity to facilitate lipid utilization in mice, and *CLSTN3B* marks human thermogenic fat [5]. However, the relevance of *CLSTN3B* in human white fat remains unknown. We thus aimed to examine its transcript profile in human WAT and associate it with metabolic status in individuals.

We included a total of 210 participants with body mass index (BMI) ranging from 18.0 to 65.4 kg/m^2^ in the present study. Anthropometric and biochemical parameters were measured, and paired samples of abdominal subcutaneous adipose tissue (SAT) and visceral adipose tissue (VAT) were obtained from patients who underwent cholecystectomy or bariatric surgery. We first determined the transcript profile of the *CLSTN3B* gene with quantitative polymerase chain reaction (PCR) and then performed gene co-expression analysis combined with RNA sequencing data of these samples. The clinical relevance of novel variants occurred at the first unique exon of the *CLSTN3B* gene was lastly screened in these individuals.

**Figure 1:**
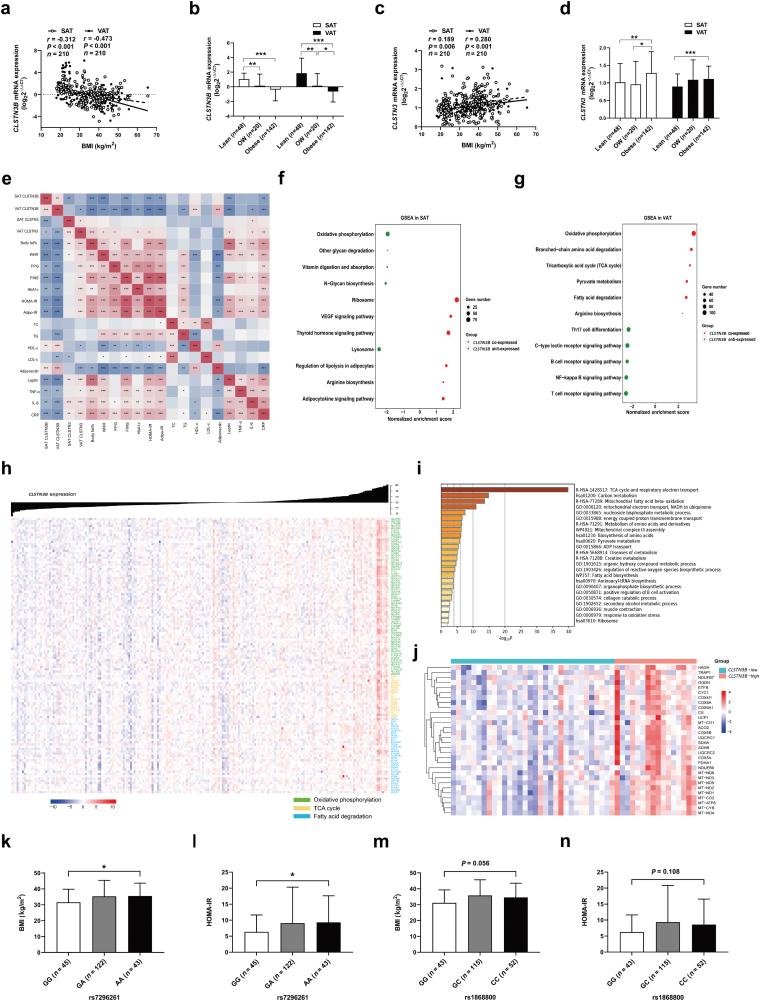
Transcript profile and potential functional roles of *CLSTN3B* gene in human adipose tissue. (a−d) Correlation of *CLSTN3B* (a) and *CLSTN3* (c) mRNA expression in abdominal SAT and VAT samples with BMI in 210 participants, and comparison of *CLSTN3B* (b) and *CLSTN3* (d) expression in SAT and VAT samples between the lean, OW, and obese groups. (e) Spearman correlation analysis of SAT and VAT *CLSTN3B* expression with metabolic parameters. (f and g) Spearman correlation analysis was performed between mRNA expressions of *CLSTN3B* and other genes in abdominal adipose tissue from 210 participants. Enriched gene sets significantly correlated with *CLSTN3B* expression were determined through GSEA, and enriched functional pathways in SAT (f) and VAT (g) samples were shown. (h) Heatmap showing the leading enriched gene sets in VAT, including oxidative phosphorylation, tricarboxylic acid cycle (TCA cycle), and fatty acid degradation. (i and j) DEGs between the *CLSTN3B*-high and *CLSTN3B*-low VAT samples from 48 lean participants were identified by the criteria fold change > 1.2 or < 0.833, and FDR < 0.05. Functional enrichment analysis of DEGs (i), and heatmap showinggenes predominantly enriched in TCA cycle and respiratory electron transport (j). (k and l) BMI (k) and HOMA-IR (l) were compared between the GG and AA genotypes for rs7296261. (m and n) BMI (m) and HOMA-IR (n) between the GG and CC genotypes for rs1868800. Statistical values are described in each graph. Variables were compared between groups using unpaired Student’s *t*-test (normal distribution) or Wilcoxon rank-sum test for non-normally distributed data. Data are expressed as mean ± SD. ^*^P < 0.05, ^**^P < 0.01, and ^***^P < 0.001 were used for the comparison.

Firstly, we analyzed the distribution of RNA sequencing reads at the *CLSTN3B* locus, and revealed that this novel gene exhibited a distinctive expression pattern in WAT between individuals: it was highly expressed in some WAT samples and extremely low in others (Supplementary Fig. S1a). Genotype-Tissue Expression database showed that *CLSTN3B* is exclusively expressed in human adipose tissue, in comparison to other metabolic tissues (Supplementary Fig. S1b). We performed quantitative PCR analy­sis of paired SAT and VAT samples from 210 participants who were lean (*n* = 48), overweight (OW, *n* = 20), and obese (*n* = 142) to determine the transcript profile of the *CLSTN3B* gene. The clinical characteristics of study participants are shown in Supplementary Table S1. *CLSTN3B* mRNA expression in SAT and VAT samples was inversely correlated with BMI (SAT, *r* = −0.312, *P* < 0.001; VAT, *r* = −0.473, *P* < 0.001; Fig. 1a), and *CLSTN3B* expression significantly decreased in both fat depots from the lean group to the obese group (Fig. 1b). By contrast, transcript level of *CLSTN3* in SAT samples was positively correlated with BMI (*r* = 0.189, *P* = 0.006), and this association was also observed in VAT samples (*r* = 0.280, *P* < 0.001; Fig. 1c). The *CLSTN3* expression was significantly higher in SAT and VAT samples from obese participants than those from lean participants (Fig. 1d). Furthermore, *CLSTN3B* expression was inversely correlated with *CLSTN3* expression both in SAT and VAT samples (SAT, *r* = −0.254, *P* < 0.001; VAT, *r* = −0.248, *P* < 0.001; Supplementary Fig. S1c and d).

Next, we examined whether *CLSTN3B* expression in WAT relates to obesity-associated metabolic status. Spearman correlation analysis was performed between adipose tissue *CLSTN3B* expression and metabolic variables in these individuals. *CLSTN3B* expression in SAT and VAT samples was inversely correlated with several metabolic characteristics of obesity and adipose tissue, including body fat%, waist-to-hip ratio, circulating levels of triglyceride (TG), adipokine leptin, and inflammatory cytokines [tumor necrosis factor-α (TNF-α), interleukin-6 (IL-6), and C-reactive protein (CRP)], and it was positively associated with circulating concentrations of high-density lipoprotein cholesterol (HDL-C) and adiponectin. In addition, *CLSTN3B* expression was inversely correlated with certain parameters of whole-body and adipose glucose metabolism, including fasting plasma glucose (FPG), fasting insulin (FINS), glycosylated hemoglobin A1c (HbA1c), adipose tissue insulin resistance (Adipo-IR), and homeostasis model assessment of insulin resistance (HOMA-IR). Interestingly, the correlation of *CLSTN3B* expression with metabolic variables was stronger in VAT than in SAT samples. Meanwhile, *CLSTN3* expression in SAT and VAT samples was significantly correlated with some metabolic characteristics, which was mostly opposite to that of the *CLSTN3B* gene (Fig. 1e and Supplementary Table S2). Moreover, when adjusted for BMI, the correlation of *CLSTN3B* expression with FPG, FINS, HbA1c, HOMA-IR, Adipo-IR, TG, and LDL-C retained statistically significant only in VAT samples (Table 1).

**Table 1.** Correlation analysis of *CLSTN3B* expression in SAT and VAT samples with metabolic parameters.

Variables	*CLSTN3B* mRNA expression	
	SAT *r*_BMI_	VAT *r*_BMI_
Body fat%	−0.063	−0.056
WHR	−0.249^***^	−0.099
FPG (mmol/L)	−0.107	−0.212^**^
FINS (mU/L)	−0.119	−0.176^*^
HbA1c (%)	−0.099	−0.169^*^
HOMA-IR (mmol/L × mU/L)	−0.113	−0.213^**^
Adipo-IR (mmol/L × mU/L)	−0.102	−0.203^**^
TC (mmol/L)	0.008	−0.116
TG (mmol/L)	−0.065	−0.223^**^
HDL-C (mmol/L)	0.151^*^	0.244^***^
LDL-C (mmol/L)	−0.017	−0.174^*^
Adiponectin (mg/L)	0.147^*^	0.361^***^
Leptin (ng/mL)	−0.100	−0.085
TNF-α (pg/mL)	−0.076	−0.027
IL-6 (pg/mL)	−0.048	−0.091
CRP (mg/L)	−0.073	−0.047

Note: *r*_BMI_, partial correlation coefficient adjusted for BMI.

Abbreviations: WHR, waist-to-hip ratio; FPG, fasting plasma glucose; FINS, fasting insulin; HbA1c, glycated hemoglobin A1c; HOMA-IR, homeostasis model assessment of insulin resistance; Adipo-IR, adipose tissue insulin resistance; TC, total cholesterol; TG, triglyceride; HDL-C, high-density lipoprotein cholesterol; LDL-C, low-density lipoprotein cholesterol; TNF-α, tumor necrosis factor-α; IL-6, interleukin-6; CRP, C-reactive protein.

^*^P < 0.05, ^**^P < 0.01, and ^***^P < 0.001.

Further, we performed *CLSTN3B* co-expression analysis to predict its functional processes in SAT and VAT samples. Since *CLSTN3B* has not been updated in the human hg38 genome, here we combined the quantitative PCR and transcriptional data of these samples to analyze the correlation between the expressions of *CLSTN3B* and other genes. Gene Set Enrichment Analysis (GSEA) was performed to identify *CLSTN3B*-associated gene sets and pathways enriched in the ranked gene lists. Genes co-expressed with *CLSTN3B* in SAT samples showed a significant enrichment in pathways related to ribosome and vascular endothelial growth factor signaling pathway, and genes anti-expressed with *CLSTN3B* were enriched in pathways related to lysosome and so on (Fig. 1f). However, gene sets co-expressed with *CLSTN3B* expression in VAT samples were enriched in mitochondrial energy metabolism, such as oxidative phosphorylation, branched-chain amino acid degradation, tricarboxylic acid cycle (TCA cycle), pyruvate metabolism, fatty acid degradation, and arginine biosynthesis, and gene sets negatively anti-expressed with *CLSTN3B* expression were enriched in inflammatory activities (Fig. 1g). Additionally, as shown in the enrichment plots and heatmap of the leading gene sets, *CLSTN3B*-related enhancement of mitochondrial fundamental activities occurred, and expression levels of genes involved in oxidative phosphorylation, TCA cycle, and fatty acid degradation were upregulated with the increase of *CLSTN3B* expression in human VAT (Supplementary Fig. S2a, Fig. 1h). Moreover, as shown in the scatter plot, expression levels of beige/brown adipocyte marker genes (*PPARGC1A*, *CIDEA*, and *UCP1*) were positively correlated with *CLSTN3B* expression in VAT samples (Supplementary Fig. S2b).

Given that obesity status substantially influenced gene expressions in human VAT, we restrictively explored gene signatures associated with *CLSTN3B* in lean individuals. There was no significant correlation between VAT *CLSTN3B* expression and BMI (*r* = 0.207, *P* = 0.158; Supplementary Fig. S2c). Differential expression analysis revealed 132 differentially expressed genes (DEGs), including 118 upregulated and 14 downregulated genes, in *CLSTN3B*-high VAT samples (*n* = 16) compared with the *CLSTN3B*-low samples (*n* = 32). In the functional enrichment analysis, DEGs were mainly enriched in TCA cycle and respiratory electron transport, mitochondrial fatty acid oxidation, and several other metabolic processes (Fig. 1i). Indeed, *CLSTN3B*-high VAT samples showed higher expression levels of mitochondrial genes, such as the most enriched biological process, TCA cycle and respiratory electron transport, than the *CLSTN3B*-low VAT samples (Fig. 1j).

Finally, we sequenced the unique protein-coding region of *CLSTN3B* in all participants to identify its genetic variation and associated the variants with metabolic traits. Three variants were found to be located within this region, including rs7296261 (g.398G>A, p.R133Q), rs1868799, and rs1868800 (it undergoes a missense mutation p.R224S if the allele of rs1868799 and rs1868800 is A and C, respectively). Genotyping for rs7296261 resulted in 45 GG, 122 GA, and 43 AA genotype carriers. Compared with GG genotype carriers, AA genotype carriers had significantly higher BMI (31.6 ± 8.3 vs. 35.5 ± 8.1, *P* < 0.05; Fig. 1k), which was in line with our previous work [4]. In addition, mean HOMA-IR was significantly higher in AA genotype carriers than in GG genotype carriers (6.4 ± 5.3 vs. 9.3 ± 8.3, *P* < 0.05; Fig. 1l). We further found that changes in BMI and HOMA-IR did not differ between the two genotypes for rs1868799 (Supplementary Fig. S3a and b). Genotyping for rs1868800 divided participants into three groups, namely GG (*n* = 43), GC (*n* = 115), and CC (*n* = 52) genotypes. Compared with GG genotype carriers, CC genotype carriers showed an increasing trend in BMI (31.1 ± 8.2 vs. 34.6 ± 9.0, *P* = 0.056; Fig. 1m), as well as in HOMA-IR (6.3 ± 5.4 vs. 8.6 ± 8.0, *P* = 0.108; Fig. 1n).

To our knowledge, this study is the first to report that *CLSTN3B* expression in human WAT correlates with BMI and various metabolic parameters. *CLSTN3B* co-expression analysis in VAT samples shows that the transcript profile of this gene also associates with a mitochondrial gene program. Our data provide further insights into the potential function of the *CLSTN3B* gene in human adipose tissue and obesity in addition to its roles in thermogenic fat in mice. The mouse adipocyte-specific *Clstn3b* gene encodes a protein, CLSTN3β, localized at endoplasmic reticulum (ER), and it partly drives the secretion of the neurotrophic factor S100B to maintain innervation of thermogenic adipose tissue [3]. Another independent study showed that CLSTN3β localizes to ER-lipid droplet contact sites through a conserved hairpin-like domain; it associates with the cell death-inducing DNA fragmentation factor 45 (DFF45)-like effector (CIDE) proteins to enforce adipocyte multilocularity and facilitate lipid utilization of thermogenic adipocytes in mice; *CLSTN3B* is a selective marker of thermogenic fat in humans [5]. Thermogenic (brown and beige) adipocytes have a remarkable ability to oxidize fatty acids, whereas white adipocytes residing in WAT are specialized for energy storage and mobilization [6]. In the present study, the strong association between *CLSTN3B* expression and mitochondrial gene program in white fat challenges underlying mechanisms. ER-mitochondria contacts are involved in the regulation of lipid metabolism and mitochondrial dynamics [7]. Recently, Combot *et al*. demonstrated that seipin is localized to ER-lipid droplet contact sites under lipid loading, while seipin association with ER-mitochondria contact sites is stimulated under a fasting state and thus promotes mitochondria oxidation in adipocytes [8]. Therefore, more studies are needed to decipher the potential role of CLSTN3β on mitochondrial function.

Outstanding aspects of WAT are its anatomical location and function. SAT depot comprises the majority of body fat, while small VAT depot is often considered more pernicious, and VAT hypertrophy is associated with insulin resistance and metabolic dysfunction [9]. Our present work shows that the *CLSTN3B* transcript in VAT samples is more relevant to clinical parameters than that in SAT samples. The correlation between *CLSTN3B* expression and key metabolic parameters adjusting for BMI retains statistically significant only in VAT samples. *CLSTN3B* co-expression analysis in VAT samples reveals that this gene may function in adipose homeostasis by influencing mitochondrial activities. Mechanistic investigations are warranted to verify this novel role of *CLSTN3B* in mitochondrial signature. Previous study has shown that VAT depot has stronger mitochondrial activity and higher thermogenic gene expression than SAT [10]. Furthermore, a single-cell sequencing study identified a potentially visceral thermogenic adipocyte subpopulation (hAd6) characterized by higher mitochondrial intensity and increased expression of mitochondrial and thermogenic genes [11]. High expression level of *CLSTN3B* in some VAT samples and the strong association with expression levels of mitochondrial genes suggest that VAT depot may predispose to have a highly active metabolic characteristic under specific cues. Adipose tissue harbors heterogeneous cell populations, including progenitor cells, adipocytes, and immune cells, and cell types with high *CLSTN3B* expression in human fat need to be further elucidated at the ­single-cell resolution. Given the adipose-specific expression of the *CLSTN3B* gene and its potential role in regulating mitochondrial homeostasis, adipose-targeted gene therapy may be a novel approach to treat obesity and associated metabolic disorders.

Many genetic variants have been associated with obesity and type 2 diabetes, and the majority are located in non-coding regions. Only a minority of genetic variants are located within ­protein-coding regions, which marks phenotypic impact by affecting protein function [12]. We have previously shown that an intronic variant rs7296261 at the *CLSTN3* locus associated with obesity risk and high *CLSTN3* expression in human adipose tissue [4]. This variant is simultaneously localized to the first exon of the *CLSTN3B* gene, and here, we screen 210 individuals and observe three common variants within this unique region. Among these loci, rs7296261 affecting the R133Q mutant is the only variant that is linked to metabolic phenotypes, namely, AA genotype carriers have higher BMI and accelerated insulin resistance than GG genotype carriers. This mutant is located within the hairpin-like domain of the CLSTN3β protein that mediates its localization to other organelles, and it may have an impact on protein function. The variant rs1868800 affecting the R224S mutant has a highly homologous genetic variation with rs7296261 although they are spatially distant, and this mutant is located within a disordered region linking the hairpin domain to the transmembrane domain of the CLSTN3β protein. In this study, we have a better understanding of the genetic basis of the *CLSTN3B* gene, which may bring implications for the functional impact of protein mutations. More interestingly, the transcript level of *CLSTN3* in human WAT is negatively correlated with *CLSTN3B* expression. In addition, the two genes are inversely correlated with some metabolic parameters. We believe that underlying cues may be involved in the expression pattern and biological function of the two genes, and the variant rs7296261 may be one of the regulators.

Our study has several strengths. First, this study is the first to analyze the transcript profile of the *CLSTN3B* gene in human WAT and associate its level with metabolic status in individuals. Second, the strong correlation between *CLSTN3B* transcript and expression levels of mitochondrial genes highlights its potential function in human VAT, and this brings a great implication for following mechanistic studies. Finally, the protein-coding variant of *CLSTN3B* is linked to metabolic phenotypes and may provide cues for exploring underlying mechanisms. However, this study also has a few limitations. This study is unable to clarify the causal relationship between the *CLSTN3B* transcript profile in human WAT and the progression of obesity. The exact effect of the *CLSTN3B* gene on obesity warrants further confirmation in large-scale prospective studies and mechanistic studies.

In conclusion, we provide a novel observation on the transcript profile of *CLSTN3B* in human adipose tissue and highlight its associations with mitochondrial gene program and metabolic status. These findings may contribute to the development of adipose-­specific *CLSTN3B* upregulation to improve metabolic dysfunction.

## Supplementary Material

load037_suppl_Supplementary_Material

## Data Availability

All study data are included in the article and/or supplementary information. Materials and reagents are available upon reasonable request.
