# Supplementary material for: Transcript profile of CLSTN3B gene in human white adipose tissue is associated with obesity and mitochondrial gene program
Source: Life Metab. 2023 Sep 14;2(6):load037. doi: 10.1093/lifemeta/load037 (PMC11748976; doi:10.1093/lifemeta/load037)

**Supplementary Methods**

**Study participants**

A total of 210 participants (45 men and 165 women; BMI range, 18.0–65.4 kg/m^2^; age range, 16.8–64.6 years) were recruited in the present study. Paired samples of abdominal subcutaneous adipose tissue (SAT) and visceral adipose tissue (VAT) were obtained from patients who underwent cholecystectomy or bariatric surgery at Shanghai Jiao Tong University Affiliated Sixth People’s Hospital, Shanghai, China from July 2019 to August 2020. They were categorized according to WHO BMI classification: a BMI between 25.0 kg/m² and 29.9 kg/m² is considered overweight (OW); a BMI of 30.0 kg/m² or higher is considered obese (1). Patients who had severe liver or kidney dysfunction, thyroid dysfunction, or malignant tumors, or received insulin treatment were excluded from this study. Adipose tissue samples were collected during the surgery, cut into small pieces, and frozen in liquid nitrogen immediately after excision. This study was approved by the Ethics Committee of Shanghai Jiao Tong University Affiliated Sixth People’s Hospital. This study was conducted in accordance with the Declaration of Helsinki, and written informed consents were obtained from participants after the purpose of the study was explained to them.

**Anthropometric and biochemical measurements**

The weight, height, systolic blood pressure, diastolic blood pressure, waist circumference, and hip circumference of each participant were measured as previously reported (2). Fat mass was measured using an MC-780MA total body composition analyzer (Tanita, Tokyo, Japan). BMI was calculated as the weight in kilograms divided by the squared height in meters. Waist-to-hip ratio (WHR) was calculated by dividing the waist circumference by the hip circumference. Venous blood samples were collected from participants after 10 h overnight fasting. The levels of fasting plasma glucose (FPG), glycosylated hemoglobin A1c (HbA1c), fasting insulin (FINS), serum lipid profiles [including triglyceride (TG), total cholesterol, high-density lipoprotein cholesterol (HDL-C), and low-density lipoprotein cholesterol (LDL-C)], and C-reactive protein (CRP) were determined using previously described methods (3). Non-esterified fatty acid (NEFA) levels were measured using LabAssay^TM^ NEFA (FUJIFILM Wako, Osaka, Japan). Whole-body insulin sensitivity was measured using the homeostasis model assessment of insulin resistance (HOMA-IR): FPG (mmol/L) × FINS (mU/L)/22.5 (4). Adipose tissue insulin resistance (Adipo-IR), a good predictor of insulin resistance in adipose tissue, was calculated as follows: NEFA (mmol/L) × FINS (mU/L) (5).

**Measurements of serum adipokines and cytokines**

Serum adiponectin levels were quantified using a latex particle-enhanced immunoturbidimetric assay (Antibody and Immunoassay Services, University of Hong Kong). The intra- and inter-assay variations in adiponectin levels were 3.13% and 6.85%, respectively. Serum leptin levels were quantified using an enzyme-linked immunosorbent assay (R&D Systems, Minneapolis, MN, USA) with intra- and inter-assay variations of 3.27% and 6.59%, respectively. Serum concentrations of tumor necrosis factor-α (TNF-α) and interleukin-6 (IL-6) were determined using chemiluminescence (TNF-α, Siemens, Berlin, Germany; IL-6, Roche, Mannheim, Germany).

**Real-time quantitative PCR**

Total RNA was extracted from adipose tissue samples using an RNeasy Mini Kit (Qiagen, Hilden, Germany). RNA integrity was evaluated in an Agilent 2100 Bioanalyzer (Agilent Technologies, Santa Clara, CA, USA). RNA (1 μg) was reverse transcribed into cDNA using the GoScript™ Reverse Transcription System (Promega, Madison, WI, USA). Gene expression was assessed through real-time quantitative PCR using the AceQ qPCR SYBR Green Master Mix (Vazyme, Nanjing, China) on a LightCycler® 480 Real-Time PCR system (Roche, Mannheim, Germany). Quantitative gene expression levels were normalized to the *RPLP0* gene using the 2^-∆∆CT^ method. The primers used are listed in Supplementary Table S3.

**RNA sequencing**

Strand-specific RNA sequence libraries were generated with the VAHTS Total RNA Sequence Library Prep Kit (Vazyme, Nanjing, China). RNA sequencing was then performed on the Illumina NovaSeq 6000 platform (Illumina, San Diego, CA, USA). Library construction and sequencing work were performed by Sinotech Genomics Corporation (Shanghai, China). After the paired-end sequence, files (fastq) were mapped to the human hg38 genome, and gene abundance was expressed as fragments per kilobase of exon per million reads mapped (FPKM).

**Gene expression data analysis**

Spearman correlation analysis was performed between the expression of *CLSTN3B* (log_2_-transformed *CLSTN3B*, from quantitative PCR) and other genes (FPKM, from RNA sequencing) in both SAT and VAT samples of 210 participants, and gene set enrichment analysis (GSEA) was conducted using the WEB-based Gene SeT AnaLysis Tool kit (WebGestalt, http://www.webgestalt.org/) (6). Differentially expressed genes (DEGs) between *CLSTN3B*-high and *CLSTN3B*-low VAT samples grouped from 48 lean participants were identified using EBSeq algorithms in accordance with the following criteria: fold change > 1.2 or < 0.833, and false discovery rate (FDR) < 0.05; functional enrichment analysis was performed using Metascape (https://metascape.org/) (7).

**Genetic variants and genotyping**

Genomic DNA was extracted from adipose tissue samples using the Fast DNA Tissue Kit (Qiagen, Hilden, Germany) in accordance with the manufacturer’s protocol. The purity and quantity of the DNA samples were assessed using a NanoDrop spectrophotometer (Thermo Fisher Scientific, Waltham, MA, USA). Primer sequences for PCR amplification covering the unique exon-1 of the *CLSTN3B* gene were as follows: forward, 5ʹ-GTGTGACTGCTGGTCTCTGT-3ʹ and reverse, 5ʹ- GTCATGAACAGCCCTATTCG-3ʹ. Candidate variants (rs7296261, rs1868799, and rs1868800) were identified through Sanger sequencing following PCR amplification. For quality control, resequencing validation of randomly selected individuals was performed to confirm the genotyping results. *CLSTN3B* variants in 210 participants were genotyped, and obesity-associated metabolic parameters were compared between the two genotypes.

**Statistical analysis**

Statistical analyses were performed using SPSS 22.0 and GraphPad Prism 8.0. The normality of the data distribution was tested using the Kolmogorov–Smirnov test. Data with a normal distribution are expressed as mean ± standard deviation (SD), whereas skewed data are presented as median (interquartile range). Categorical variables were presented as numbers (percentages). Gene expression data with skewed distribution were logarithmically transformed to ensure normality before the analyses. Variables were compared between groups using unpaired Student’s *t*-test (normal distribution) or Wilcoxon rank-sum test for non-normally distributed data. The relationships between variables were evaluated through Spearman correlation analysis. A two-tailed *P*-value < 0.05 was considered to indicate statistical significance.

**References**

[1] Obesity: preventing and managing the global epidemic. Report of a WHO consultation. World Health Organ Tech Rep Ser 2000;894:i-xii, 1-253

[2] Lu J, Ma X, Zhou J, et al. Association of Time in Range, as Assessed by Continuous Glucose Monitoring, With Diabetic Retinopathy in Type 2 Diabetes. Diabetes Care 2018;41:2370-2376

[3] He X, Hu X, Ma X, et al. Elevated serum fibroblast growth factor 23 levels as an indicator of lower extremity atherosclerotic disease in Chinese patients with type 2 diabetes mellitus. Cardiovasc Diabetol 2017;16:77

[4] Xu Y, Ma X, Pan X, et al. Correlations between serum concentration of three bone-derived factors and obesity and visceral fat accumulation in a cohort of middle aged men and women. Cardiovasc Diabetol 2018;17:143

[5] Rosso C, Kazankov K, Younes R, et al. Crosstalk between adipose tissue insulin resistance and liver macrophages in non-alcoholic fatty liver disease. J Hepatol 2019;71:1012-1021

[6] Liao Y, Wang J, Jaehnig EJ, Shi Z, Zhang B. WebGestalt 2019: gene set analysis toolkit with revamped UIs and APIs. Nucleic Acids Res 2019;47:W199-w205

[7] Zhou Y, Zhou B, Pache L, et al. Metascape provides a biologist-oriented resource for the analysis of systems-level datasets. Nat Commun 2019;10:1523

**Supplementary Tables**

| **Supplementary Table S1** Anthropometric and clinical characteristics of study participants. | | | | |
| --- | --- | --- | --- | --- |
| Variables | Lean (*n* = 48) | Overweight (*n* = 20) | Obese (*n* = 142) | *P* value  for trend |
| Male, *n* (%) | 9 (18.8%) | 4 (20.0%) | 32 (22.5%) | - |
| Age (years) | 41.9 ± 10.1 | 43.1 ± 11.2 | 31.4 ± 8.1 | <0.001 |
| BMI (kg/m^2^) | 22.1 (20.9−23.0) | 27.7 (26.0−28.6) | 39.2 (34.0−43.2) | <0.001 |
| Waist circumference (cm) | 80.3 (75.3−85.0) | 96.3 (92.0−100.9) | 120.0 (111.0−130.0) | <0.001 |
| WHR | 0.86 (0.81−0.90) | 0.95 (0.89−0.97) | 0.99 (0.94−1.03) | <0.001 |
| Body fat% | 27.5 (23.7−30.9) | 37.5 (33.7−40.9) | 50.0 (44.1−55.1) | <0.001 |
| SBP (mmHg) | 120.0 (115.0−128.0) | 130.5 (108.0−142.0) | 130.0 (122.0−140.0) | <0.001 |
| DBP (mmHg) | 74.0 (70.0−82.0) | 81.5 (75.0−89.5) | 84.0 (76.0−91.0) | <0.001 |
| FPG (mmol/L) | 5.1 (4.7−5.3) | 5.3 (4.7−6.2) | 5.7 (5.2−6.7) | <0.001 |
| HbA1c (%) | 5.4 (5.3−5.7) | 6.1 (5.4−6.4) | 5.9 (5.5−6.7) | <0.001 |
| FINS (mU/L) | 7.0 (4.4−9.9) | 13.3 (7.2−22.4) | 30.2 (20.6−40.6) | <0.001 |
| HOMA-IR (mmol/L*mU/L) | 1.5 (1.0−2.2) | 3.5 (1.9−5.9) | 7.8 (5.4−12.2) | <0.001 |
| TC (mmol/L) | 4.6 ± 0.7 | 5.2 ± 1.2 | 5.0 ± 0.9 | 0.014 |
| TG (mmol/L) | 1.3 (0.8−1.6) | 1.6 (1.2−2.1) | 1.7 (1.2−2.3) | <0.001 |
| HDL-C (mmol/L) | 1.2 (1.0−1.6) | 1.2 (1.0−1.5) | 1.1 (0.9−1.3) | 0.011 |
| LDL-C (mmol/L) | 2.7 ± 0.8 | 3.1 ± 1.1 | 3.0 ± 0.7 | 0.042 |
| NFFA (mmol/L) | 0.54 ± 0.29 | 0.53 ± 0.25 | 0.59 ± 0.23 | 0.311 |
| Adipo-IR (mmol/L*mU/L) | 3.2 (1.6−6.9) | 7.0 (3.3−11.7) | 16.9 (10.2−26.2) | <0.001 |
| Adiponectin (mg/L) | 6.4 (4.6−8.6) | 5.5 (3.9−7.6) | 4.4 (3.4−5.7) | <0.001 |
| Leptin (ng/mL) | 6.3 (2.5−14.5) | 13.3 (5.8−30.0) | 53.5 (31.2−81.1) | <0.001 |
| TNF-α (pg/mL) | 5.6 (4.5−6.8) | 5.4 (4.8−7.1) | 7.1 (5.6−9.0) | <0.001 |
| IL-6 (pg/mL) | 1.8 (0.8−3.9) | 1.9 (0.8−2.8) | 4.1 (2.3−7.0) | <0.001 |
| CRP (mg/L) | 0.4 (0.1−1.2) | 1.5 (0.7−2.5) | 4.9 (2.1−8.6) | <0.001 |

Data are shown as mean ± standard deviation (SD) or median (interquartile range).

Abbreviations: BMI, body mass index; WHR, waist-to-hip ratio; SBP, systolic blood pressure; DBP, diastolic blood pressure; FPG, fasting plasma glucose; HbA1c, glycated hemoglobin A1c; FINS, fasting insulin; HOMA-IR, homeostasis model assessment of insulin resistance; TC, total cholesterol; TG, triglyceride; HDL-C, high-density lipoprotein cholesterol; LDL-C, low-density lipoprotein cholesterol; NFFA, non-esterified fatty acid; Adipo-IR, adipose tissue insulin resistance; TNF-α, tumor necrosis factor-α; IL-6, interleukin-6; CRP, C-reactive protein.

| **Supplementary Table S2** Spearman correlation analysis of *CLSTN3B* expression in SAT and VAT samples with metabolic parameters. | | | | | | | | |
| --- | --- | --- | --- | --- | --- | --- | --- | --- |
| Variables | SAT *CLSTN3B* | | VAT *CLSTN3B* | | SAT *CLSTN3* | | VAT *CLSTN3* | |
|  | *r* | *P* | *r* | *P* | *r* | *P* | *r* | *P* |
| Body fat% | -0.336 | <0.001 | -0.423 | <0.001 | 0.213 | 0.002 | 0.344 | <0.001 |
| WHR | -0.398 | <0.001 | -0.334 | <0.001 | 0.181 | 0.009 | 0.273 | <0.001 |
| FPG | -0.207 | 0.003 | -0.323 | <0.001 | 0.066 | 0.341 | 0.273 | <0.001 |
| FINS | -0.305 | <0.001 | -0.397 | <0.001 | 0.107 | 0.126 | 0.222 | 0.001 |
| HbA1c | -0.208 | 0.003 | -0.286 | <0.001 | 0.117 | 0.091 | 0.271 | <0.001 |
| HOMA-IR | -0.294 | <0.001 | -0.415 | <0.001 | 0.092 | 0.189 | 0.263 | <0.001 |
| Adipo-IR | -0.280 | <0.001 | -0.393 | <0.001 | 0.090 | 0.193 | 0.226 | 0.001 |
| TC | -0.006 | 0.926 | -0.114 | 0.100 | -0.134 | 0.053 | 0.028 | 0.693 |
| TG | -0.142 | 0.040 | -0.308 | <0.001 | -0.024 | 0.725 | 0.118 | 0.088 |
| HDL-C | 0.208 | 0.003 | 0.290 | <0.001 | -0.102 | 0.143 | -0.175 | 0.011 |
| LDL-C | -0.049 | 0.483 | -0.174 | 0.012 | -0.148 | 0.032 | 0.043 | 0.536 |
| Adiponectin | 0.231 | 0.001 | 0.448 | <0.001 | -0.045 | 0.522 | -0.240 | 0.001 |
| Leptin | -0.303 | <0.001 | -0.396 | <0.001 | 0.224 | 0.001 | 0.241 | 0.001 |
| TNF-α | -0.186 | 0.008 | -0.187 | 0.008 | 0.104 | 0.141 | 0.205 | 0.003 |
| IL-6 | -0.182 | 0.010 | -0.264 | <0.001 | 0.261 | <0.001 | 0.258 | <0.001 |
| CRP | -0.276 | <0.001 | -0.319 | <0.001 | 0.208 | 0.003 | 0.289 | <0.001 |

Data are shown as Spearman *r* correlation and corresponding *P* value.

**Supplementary Table S3** Primer sequences used for quantitative PCR analysis.

| Gene | Sequence |
| --- | --- |
| *CLSTN3B* (forward) | 5’--3’ GCCATCAGCTCTAAGGTCCG |
| \| *CLSTN3B* (reverse) \| \| --- \| | 5’--3’ CCACAATGATGAGGGTTGCG |
| *CLSTN3* (forward) | 5’--3’ TGTGGAGACCTTCAACCATGC |
| \| *CLSTN3* (reverse) \| \| --- \| | 5’--3’ CCGTTGGTTCCCTCAAAGTC |
| *RPLP0* (forward) | 5’--3’ AGCCCAGAACACTGGTCTC |
| \| *RPLP0* (reverse) \| \| --- \| | 5’--3’ ACTCAGGATTTCAATGGTGCC |

**Supplementary Figures**

**Supplementary Figure S1** Transcriptional expression pattern of *CLSTN3B* and *CLSTN3* gene in human adipose tissue and other metabolic tissues. (a and b) Distribution of RNA sequencing reads at the *CLSTN3* or *CLSTN3B* locus in human white adipose tissue (WAT) samples from our transcriptome data (a) and various metabolic tissues from the Genotype-Tissue Expression database (b). Arrows denote the first unique exon of *CLSTN3B* gene. (c and d) Correlation between *CLSTN3B* and *CLSTN3* mRNA expression in SAT (c) and VAT (d) samples from 210 participants. Statistical values are described in each graph.

**Supplementary Figure S2** Functional enrichment analysis of *CLSTN3B*-associated genes in human adipose tissue. (a) Enrichment plots showing the leading enriched gene sets in VAT samples, including oxidative phosphorylation, TCA cycle, and fatty acid degradation. (b) Representative scatter plots showing the correlation between the expression levels of *CLSTN3B* in human VAT and beige/brown adipocyte marker genes, including *PPARGC1A*, *CIDEA*, and *UCP1*. (c) Correlation of VAT *CLSTN3B* expression with BMI in a total of 48 lean participants. Statistical values are described in the graph.

**Supplementary Figure S3** *CLSTN3B* rs1868799 has no links with metabolic characteristics. (a and b) BMI (a) and HOMA-IR (b) between the GG and CC genotypes for rs1868799.

**Supplementary Figure S1**


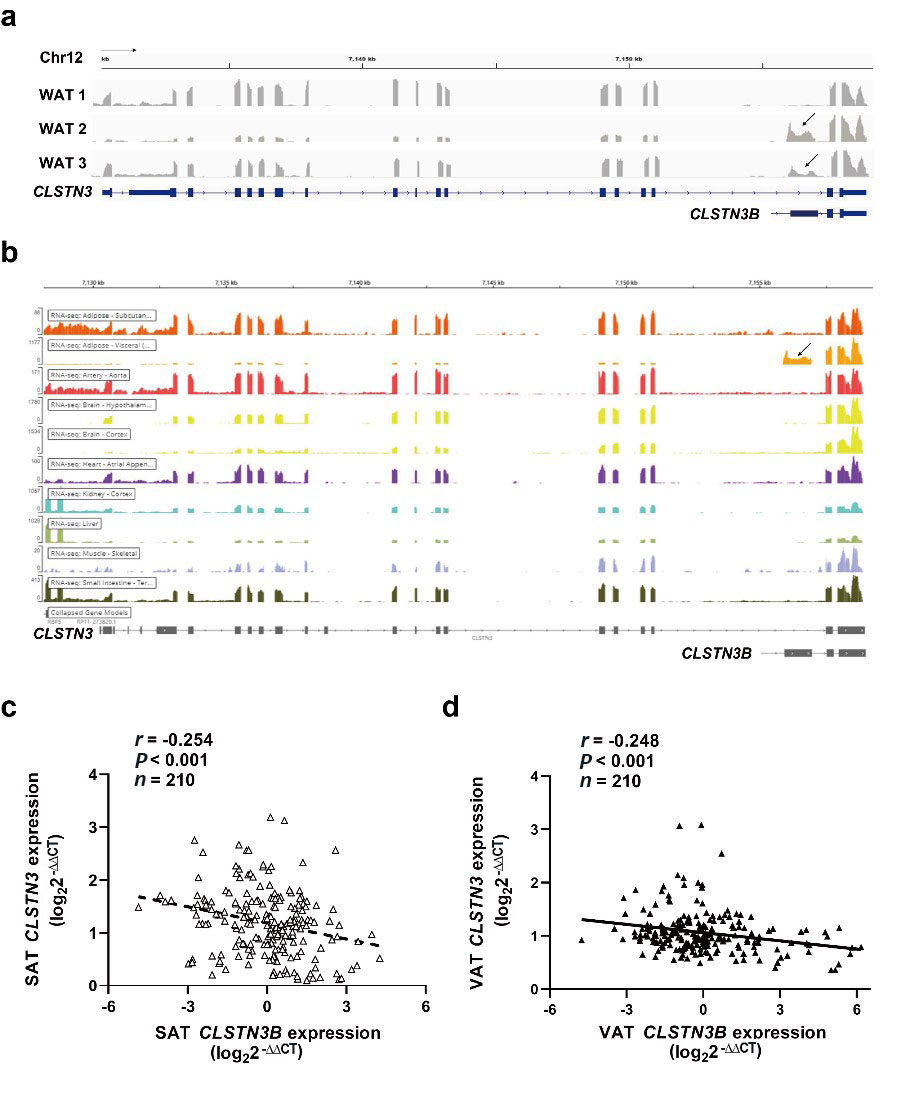


**Supplementary Figure S2**


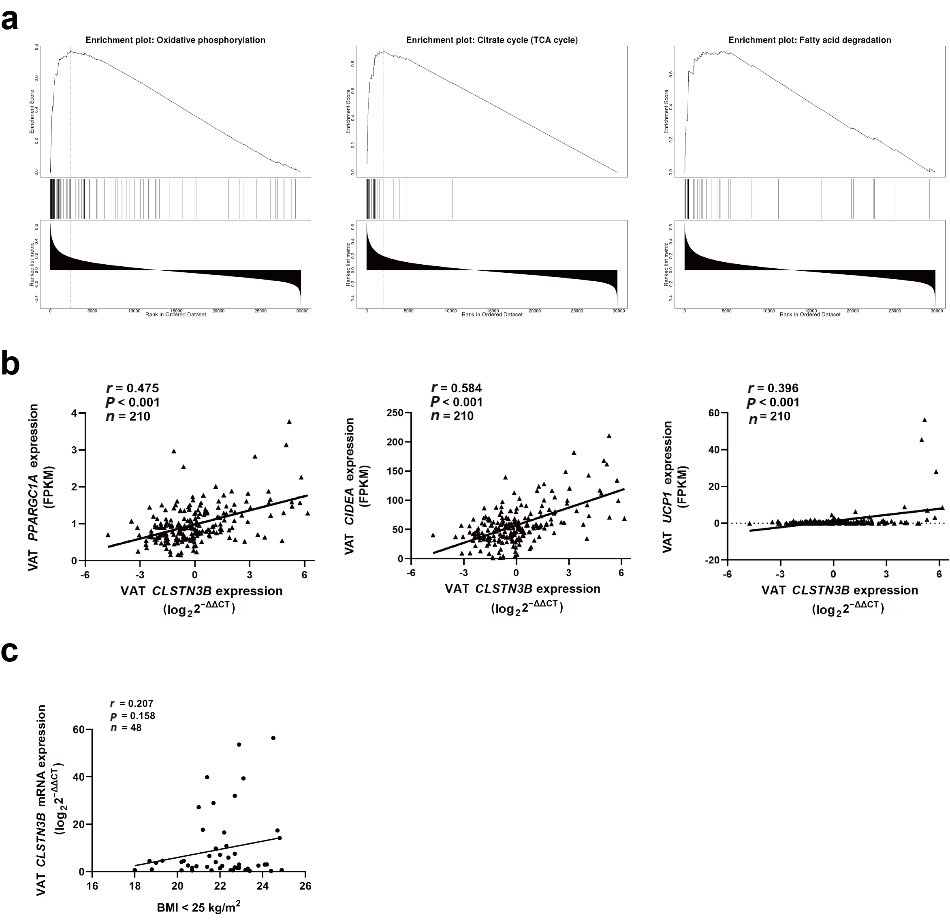


**Supplementary Figure S3**


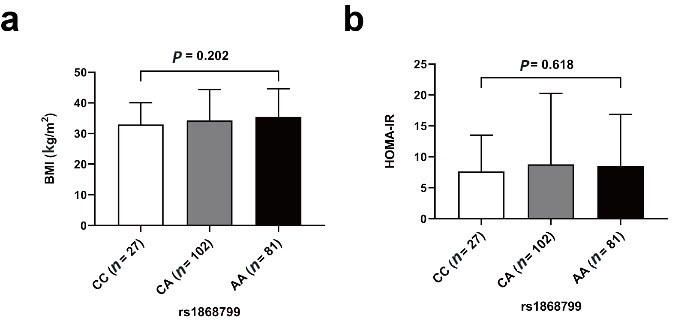

Supplement: load037_suppl_Supplementary_Material [file load037_suppl_Supplementary_Material.docx]
